# Zoonotic intestinal protozoan of the wild boars, *Sus scrofa*, in Persian Gulf’s coastal area (Bushehr province), Southwestern Iran

**DOI:** 10.14202/vetworld.2016.1047-1050

**Published:** 2016-10-06

**Authors:** Kambiz Yaghoobi, Bahador Sarkari, Majid Mansouri, Mohammad Hossein Motazedian

**Affiliations:** 1 Department of Parasitology and Mycology, School of Medicine, Shiraz University of Medical Sciences, Shiraz, Iran; 2 Basic Sciences in Infectious Diseases Research Center, Shiraz University of Medical Sciences, Shiraz, Iran

**Keywords:** Iran, Persian Gulf, protozoan, wild boars, zoonosis

## Abstract

**Aim:**

Wild boars, *Sus scrofa*, are potential reservoirs of many zoonotic diseases, and there are a possibility of transmission of the zoonotic diseases from these animals to humans and also domestic animals. This study aimed to evaluate the protozoan contamination of wild boars in the Persian Gulf’s coastal area (Bushehr Province), southwestern Iran.

**Materials and Methods:**

A total of 25 crossbred boars were collected during a course of vertebrate pest control in Bushehr province, in 2013. Samples were collected from the gastrointestinal tracts of each boar in 5% formalin, Bouin’s solution, sodium acetate-acetic acid-formalin, and polyvinyl alcohol fixatives. Fixed stool smears examined by trichrome and Ziehl–Neelsen staining.

**Results:**

Each of the 25 wild boars was infected with at least one of the intestinal protozoans. The rate of contamination with intestinal protozoan was 64% for *Balantidium coli*, 76% for *Iodamoeba* sp., 52% for *Entamoeba polecki*, 44% for *Blastocystis* sp. and 8% for *Chilomastix* sp. No intestinal coccidian was detected in studied boars when the stool samples were evaluated by Ziehl–Neelsen staining method.

**Conclusion:**

Findings of this study demonstrated that wild boars in the Persian Gulf coastal area are contaminated by many protozoans, including zoonotic protozoan, which poses a potential risk to locals as well as the domestic animals of the area.

## Introduction

Wild boars, *Sus scorfa*, are an animal species with a wide distribution. These animals are aboriginal at the most northern and central regions of Europe, Mediterranean zones and the most parts of Asia [1]. Wild boars live in west and southwest, north and north-east jungles of Iran [2]. Wild boars are omnivorous. They eat both plants and animals. They use various diets including herb roots, herb crust, seeds, small amphibious, reptiles, insect larva, carrion, and carcasses of animals. These animals are known as potential reservoirs for many parasitic diseases. Some of these diseases are limited to boars, but other diseases are transmitted to other wildlife species, domestic animals and humans [3,4].

Wild boar may pass feces containing an infectious agent to the crops fields. As farmers water the fields, the pathogens seep into the soil and contaminate the plants. People who eat the plants get the infection.

Protozoan parasites which may be transmitted from these animals to human are mainly *Balantidium coli*, *Entamoeba polecki*, *Blastocystis*, *Giardia*, *Cryptosporidium*, and *Toxoplasma gondii* [3]. Consumption of wild boars meat (by some people for therapeutic purposes or by some ethnic minorities) increases the risk of zoonosis infection transmitted from these animals to humans.

So far only a few studies have been done on parasitic infections of wild boars in Iran [5-8]. Among those few studies is Solaymani-Mohammadi *et al.*, a study which reported the protozoan infections of wild boars in Lorestan province of western Iran [5]. Infection of wild boars with *B. coli, E. polecki, Iodamoeba*, *Blastocystis*, *Entamoeba suis*, *Chilomastix mesnili*, and *Trichomonas suis* have been reported in their study [5]. This study was conducted to determine the protozoa infection of wild boars in the Persian Gulf’s coastal area (Bushehr Province), southwestern Iran.

## Materials and Methods

### Ethical approval

Ethical approval of the study was obtained from the Ethics Committee of Shiraz University of Medical Sciences, Shiraz, Iran.

### Study area

**Figure-1:**
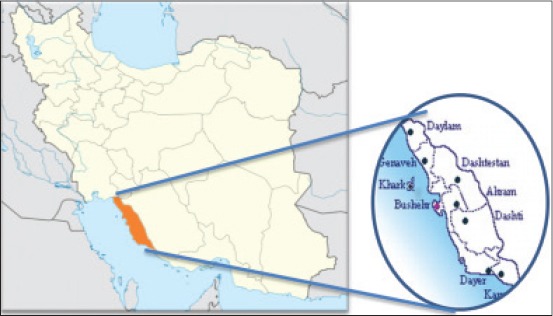
Map of Iran and study area.

Bushehr province is located in the southwest of Iran. The province has a common border with Khuzestan, Kohgiluyeh and Boyer-Ahmad and part of Fars Provinces (Figure-1). Highland nature and dense jungles in the north parts of this province and also rivers which is originated from Khuzestan province make this region to be suitable habitat for wild boars and other animal species.

### Wild boars sampling

After getting approval from the Ethics Committee of the Institute (SUMS), 25 wild boars were collected in Bushehr province, in 2013. From the total of 25 animals, 11 (44%) were male and 14 (56%) were females. Data related to gender, age of animals (on the basis of tooth shape and development) was recorded during sampling of each animal.

### Identification of protozoans

Stool samples were collected from the gastrointestinal tracts of each wild boar in 5% formalin, Bouin’s solution, sodium acetate-acetic acid-formalin, and polyvinyl alcohol fixatives. Temporary staining of stool samples with Lugol’s solution was done to find out any protozoan cysts or trophozoites. Stool samples were also examined with formalin-ethyl acetate sedimentation technique and the sedimentary materials were observed by conventional light microscope for any protozoan cyst or trophozoites. Stool smears were prepared from fixed samples, using horse serum for adhesion of specimens to slides, and carefully examined after staining with both trichrome and Ziehl–Neelsen stains.

### Statistical analysis

Collected data were entered into SPSS for Windows (Release 16). Relationships between protozoa contamination with other variables were assessed by Chi-square test. The significance level was set to 5%.

## Results

**Figure-2:**
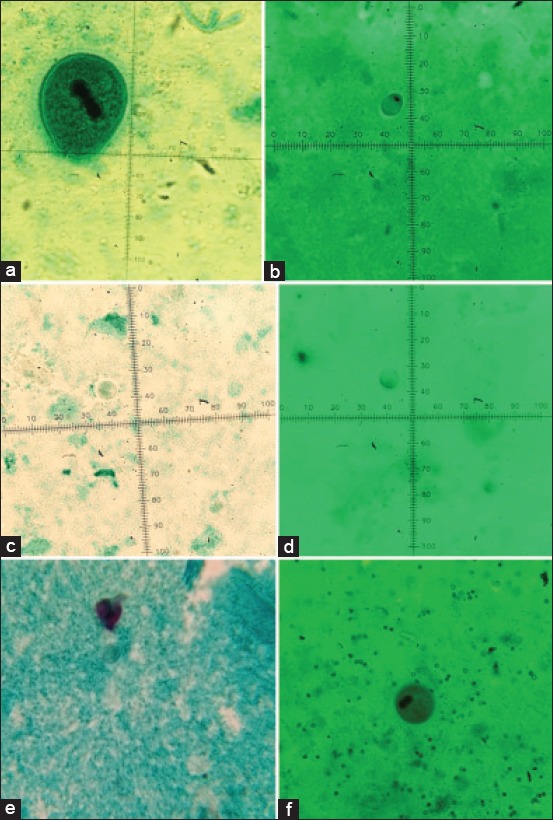
Trichrome staining of intestinal protozoan detected in the stool of wild boars. (a) *Balantidium coli* trophozoite (100×), (b) *Iodamoeba* sp. cyst (100×), (c) *Entamoeba polecki* cyst (100×), (d) *Blastocystis* sp. cyst (100×), (e) *Chilomastix* sp. cyst (100×), (f) *Balantidium coli* cyst (40×).

Each of the 25 wild boars was infected with at least one of the intestinal protozoans. The rate of contamination with intestinal protozoan was 64% for *B. coli*, 76% for *Iodamoeba* sp., 52% for *E. polecki*, 44% for *Blastocystis* sp., and 8% for *Chilomastix* sp. No intestinal coccidian was detected in the studied boars when the stool samples were evaluated by Ziehl–Neelsen staining method. Figure-2 shows a few of intestinal protozoans which have been detected in wild boars in this study.

Male boars were more infected by protozoan parasites than females (p<0.05). Table-1 shows the rate of intestinal protozoan infection in the wild boars based on sex.

**Table-1:** Rate of intestinal protozoa infection in wild boars from southwestern Iran, based on gender of animals.

Protozoa	Female	Male	Total

	N (%)	N (%)	N (%)
*B. coli*	8 (57.1)	8 (72.7)	16 (64)
*Iodamoeba* sp.	10 (71.4)	9 (81.8)	19 (76)
*E. polecki*	6 (42.8)	7 (63.6)	13 (52)
*Blastocystis* sp.	4 (28.6)	7 (63.6)	11 (44)
*Chilomastix* sp.	1 (7.1)	1 (9.1)	2 (8)

*B. coli=Balantidium coli, E. polecki=Entamoeba polecki*.

The average size of trophozoite of *B. coli* and average size of macro nucleuses were 80 µm×62.5 µm and 21.5 µ×11.5 µ, respectively. The proportion of length to the width was 1.86 µm. No significant association was found between protozoa infection and weight or age of the examined boars (χ^2^=1.64, df=3, p>0.05).

## Discussion

Wild boars are usually infected with a range of parasitic protozoan including *B. coli, E. polecki*, *Blastocystis* sp., *Giardia*, and *Cryptosporidium*. Therefore, there is a possibility of transmission of these parasites to human through consumption of contaminated water or food [3].

*B. coli* is a cosmopolitan protozoan which lives in the intestine of mammalian hosts. Wild boars and domestic pigs are considered as the main reservoirs for *B. coli* [9]. Very high prevalence rate (more than 90%) of *B. coli* has been reported from pigs in India [10].

In our study, the rate of infection with *B. coli* in wild boars was quite high (64%). The previous study in western part of Iran revealed a relatively lower (25%) prevalence rate for this protozoan [5]. Cases of human balantidiasis have been previously reported from Persian Gulf region [11]. Considering the fact that raising and breeding of domestic pigs are forbidden in Iran due to Islamic law, it can be postulated that wild boars are involved in transmission of *B. coli* to humans in this area. In view of that, wild boars may be considered as the main reservoir of *B. coli* in the region.

*E. polecki* was the third common parasite of wild boars in this study. This amoeba is best known for its infection in primates and pigs, and these animals are the main reservoirs for this protozoan. However, human infections with *E. polecki* have been reported [12,13]. Infection with *E. polecki* has also been found in sheep, goats, cattle, and wild ungulates. This parasite is considered as a nonpathogenic parasite in humans [14].

*Iodamoeba butschlii* is the protozoan parasite of human, wild boars, pigs, and monkeys [15,16]. This ameba is nonpathogenic parasite lives in human large intestine [17]. In our study, *Iodamoeba* sp. was the most common parasite of the wild boars. Lower prevalence rate (17%) of this parasite was reported from wild boars in western Iran [5].

More than 40% of studied wild boars in the current study were infected with *Blastocystis* sp. *Blastocystis* is a protozoan parasite lives in the intestine of humans and animals (e.g. dogs, cats, pigs, wild boars, and cattle) [18]. It can be found in the stools of healthy people as well as in the stools of diarrheic patients [16].

The prevalence rate of *Chilomastix* in this study is in line with Solaymani-Mohammadi’s study in western Iran [5]. *E. suis* and *T. suis* were reported from wild boars in western Iran, while in this study infection with this protozoan were not seen [5]. This may be linked to the differences in the vegetation and diet habits of these wild boars in studied regions.

## Conclusion

Findings of this study demonstrated that wild boars in the Persian Gulf’s coastal area are contaminated by many protozoans, including zoonotic ones, which poses a potential risk to locals as well as the domestic animals of the area. Moreover, the study further confirmed that previous cases of human balantidiasis in the area are more likely linked to infection of wild boars with this protozoan.
